# Characterization of the tumor microenvironment in breast cancer brain metastasis^☆^

**DOI:** 10.1016/j.heliyon.2024.e34876

**Published:** 2024-07-23

**Authors:** Jingrong Li, Nanping Lin, Shengcen Zhang, Lihong Weng, Chen Chen, Wenshi Ou, Yingping Cao

**Affiliations:** a Department of Clinical Laboratory, Fujian Medical University Union Hospital, Fuzhou, Fujian, 350001, China; b Department of Hepatopancreatobiliary Surgery, The First Affiliated Hospital of Fujian Medical University, Fuzhou, Fujian, 350001, China; c Fujian Cancer Hospital, Fuzhou, Fujian, 350001, China; d Department of Radiotherapy, Fujian Medical University Union Hospital, Fuzhou, Fujian, 350001, China

**Keywords:** Breast cancer brain metastasis, COL1A1, apCAF, Immunotherapy

## Abstract

**Objective:**

The difference in the tumor microenvironment (TME) between primary breast cancer (PBC) and breast cancer brain metastasis (BCBM) is still unknown. Herein, we present the landscape of the TME in PBC and BCBM to better understand the process of BCBM.

**Methods:**

The Gene Expression Omnibus (GEO) database was used to obtain suitable PBC and BCBM data. Hub genes that were differentially expressed between the two groups were searched. Gene Ontology (GO) and KEGG were used to define the gene's function. Single-cell data were also analyzed to determine the difference between PBC and BCBM.

**Results:**

Two datasets (GSE100534 and GSE125989) were used to search for hub genes, and 79 genes were either upregulated or downregulated between the two groups. Four hub genes (COL1A1, PDGFR, MMP3 and FZD7) were related to prognosis. GO and KEGG analyses showed that extracellular matrix and focal adhesion play major roles in the metastasis process. Another two datasets (GSE176078 and GSE186344) were enrolled for single-cell analysis. Single-cell analysis demonstrated that immune cells (66.6 %) form the main part of PBC, while cancer-associated fibroblasts (CAFs) (21.7 %) are the main component of BCBM. Immune cell proportion analysis showed that CD4+/CD8+ T cells (28.9 % and 14.3 %, respectively) and macrophages(M2) accounted for the majority of cells in PBC and BCBM, respectively. Further analysis of the classification of CAFs showed that apCAFs were significantly higher in PBC.

**Conclusions:**

This study presents the landscape of BCBM with hub gene searching and single-cell analysis. Showing the difference in the tumor/immune microenvironment of PBC and BCBM, would be beneficial to explore immunotherapy and targeted therapy for BCBM.

## 1 Introduction

Breast cancer brain metastasis (BCBM) is a refractory malignancy with a poor overall survival of less than 17.5 months [1]. Although some pathway and physiological merchandise were found in previous studies [2,3], the tumor microenvironment of BCBM still didn't well understand. Due to significant obstacles, such as the blood‒brain barrier (BBB), many treatment strategies are useless [4,5]. Further research is greatly needed for BCBM.

Cancer-associated fibroblasts (CAFs) play a critical role in the tumor microenvironment, such as tumor metabolism, immune escape and angiogenesis, in patients with metastatic tumors [[6], [7], [8]]. Even though the merchandise showed the treatment value of CAFs, CAF-targeted treatment strategies often turn out to be unsatisfactory [8]; moreover, some of the treatments accelerate tumor growth and metastasis [7]. Recently, more subgroups of CAFs have been introduced to the public, and the relationship between the immune microenvironment of tumors and CAFs has become more distinct [9], which has led to great progress in CAF-targeted treatment strategies in solid tumors, particularly in metastatic tumors [10,11].

Antigen-presenting CAFs (apCAFs) were first introduced by Elyada et al.^9^in 2019, and many studies have shown that apCAFs can directly connect and induce naïve CD4^+^ T cells into regulatory T cells, which weakens the efficacy of immunotherapy [12,13]. While some recently studies [10,11] have shown that apCAFs promote CD8^+^ T cells and enhance antitumor immunity, which provides insight into treatment strategies to enhance cancer immunotherapy, further research on apCAFs is greatly needed.

To better understand the tumor/immune microenvironment of BCBM and determine the fundamental connection between apCAFs and immunotherapy in BCBM, we conducted this study using a public database to show the connection and provide basic information for CAF-associated treatment.

## 2 Materials and methods

### 2.1 Data collection and processing

#### 2.1.1 RNA-sequencing data

Public data were collected from the Gene Expression Omnibus (GEO) database. The data should meet the following criteria: 1. Samples contain both primary and brain metastasis of breast cancer. 2. Samples were *Homo sapiens*. 3. The sample size was more than 20 for each dataset. 4. Complete data were available in GEO. Finally, two GEO datasets (GSE100534 and GSE125989) were included for further analysis; GSE38057 was included for survival analysis (Survival time was figured up according to the data of primary tumor diagnosis and the date of last information or date of death). GSE18544 (11 samples), GSE80722(10 samples) and GSE26261 (16 samples) were excluded for insufficient sample size. Due to the lack of brain metastasis data in TCGA, the TCGA database was not included in this study.

#### 2.1.2 Single-cell data

The single-cell data were collected from the GEO database. The data met the following criteria: 1. Samples contained both primary and metastatic breast cancer. 2. Samples were Homo sapiens. 3. Complete data were available in GEO. Finally, two datasets were included: GSE176078, primary breast cancer samples, and GSE186344, breast cancer brain metastasis samples.

### 2.2 Pathway enrichment analyses and hub gene detection

#### 2.2.1 Pathway enrichment analyses

Two datasets were enrolled to detect the different genes expression, absolute logFC >1 and *P*-value <0.05 were set as threshold. After identifying the upregulated and downregulated genes between primary and brain metastasis breast cancer, GO and KEGG pathway enrichment analyses were conducted by using R software. *P*-value <0.05 were set as threshold.

#### 2.2.2 Hub gene detection and survival analysis

After identifying the upregulated and downregulated genes between primary and brain metastasis breast cancer, survival analysis was conducted to detect the hub genes. *P*-value <0.05 were considered with statistical difference.

### 2.3 Correlation analysis between immune cell infiltration and hub gene expression

The correlation between immune cell infiltration and hub gene expression was assessed by CIBERSORT, CD 8+ T cells, CD4^+^ T cells, macrophages, neutrophils and myeloid dendritic cells. The thresholds of an absolute value of R greater than 0.25 and a p value less than 0.01 were set by using Person's test.

### 2.4 Single-cell analyses between primary breast cancer and breast cancer brain metastasis

The microenvironment between primary breast cancer and breast cancer brain metastasis was assessed by single-cell analysis, and R software with the Seurat package was used. The resolution was set to 1. The singleR package was used to identify the cell type with Human Primary Cell Atlas Data and Blueprint Encode Data. The proportion of each cell type was assessed by the singleR package to avoid personal subjectivity. Even though the resolution set was higher than that in previous studies, some of the cell types were hard to isolate.

To further explore the immune microenvironment between primary breast cancer and breast cancer brain metastasis, the immune cells were analyzed independently using the same method described above. CAFs were also explored independently.

## 3 Result

**Fig. 1:**
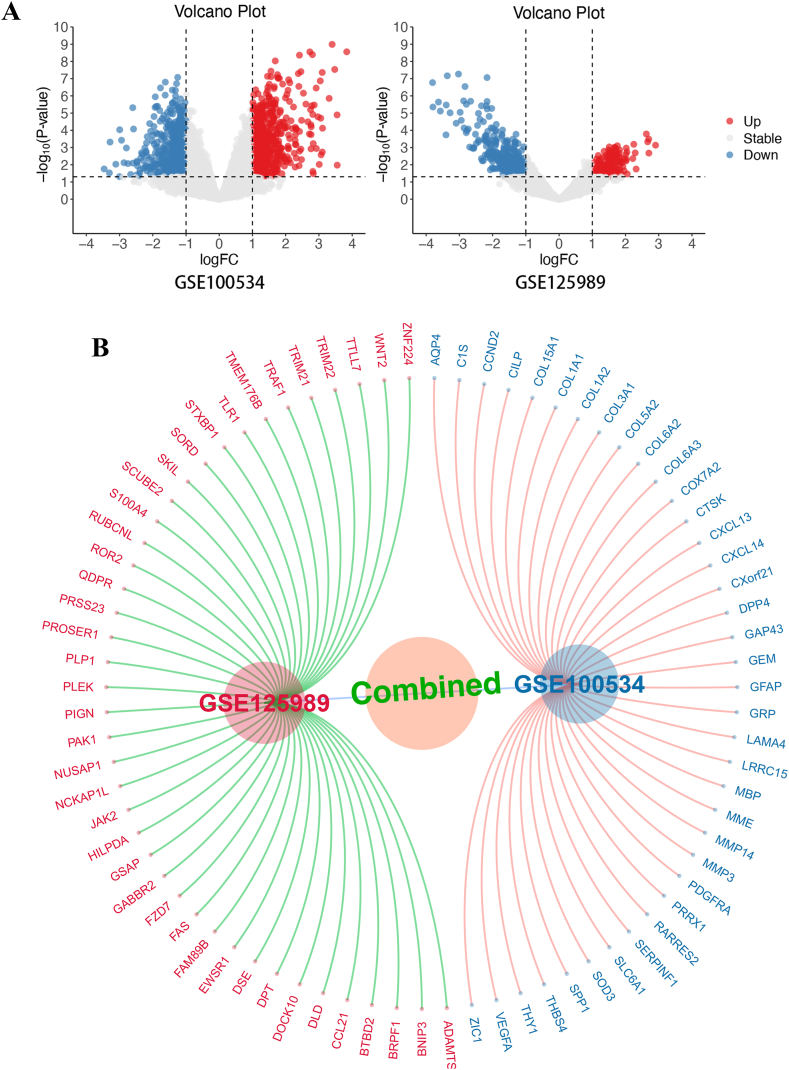

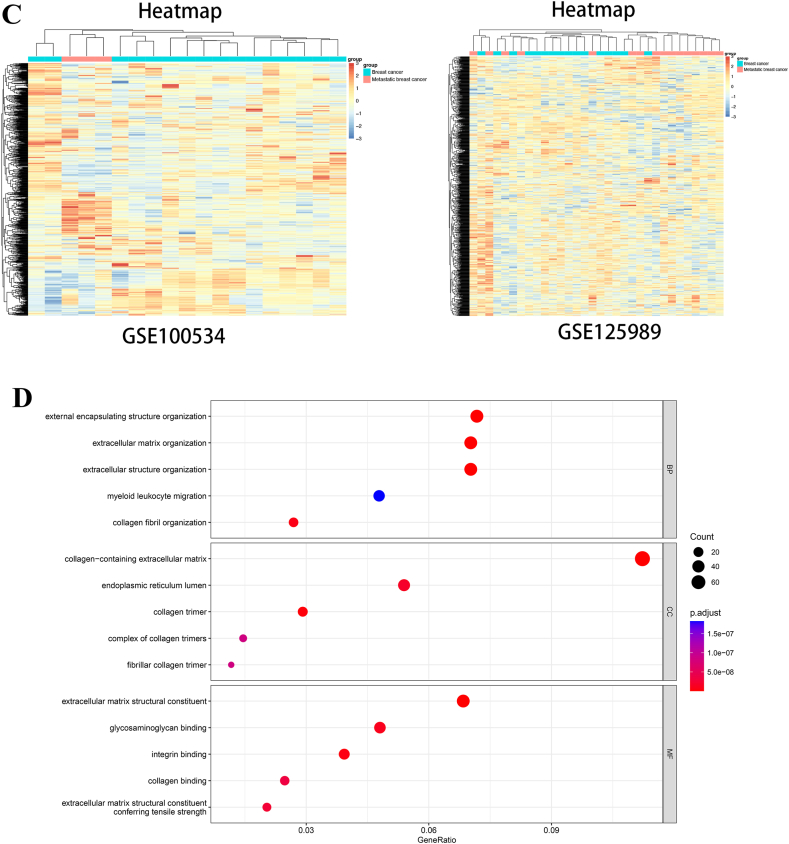

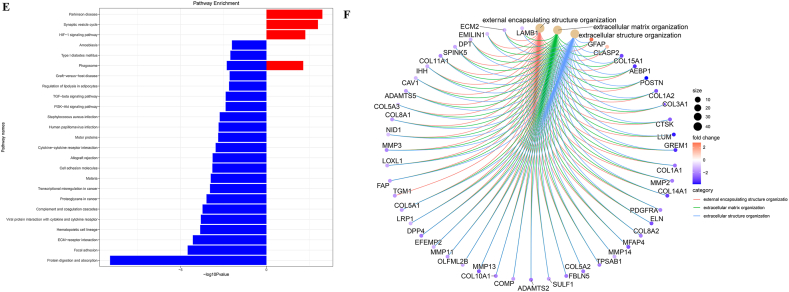
The gene expression difference between PBC and BCBM. Fig. 1A volcano plots of DEGs distribution of GSE100534 and GSE125989; Fig. 1B Upregulated of downregulated genes in two datasets; Fig. 1C Heatmap plots of DEGs of GSE100534 and GSE125989; Fig. 1D GO analysis of hub genes; Fig. 1E KEGG analysis of hub genes; Fig. 1F Circle map of hub genes and function.

After searching the GEO database, GSE100534 and GSE125989 were included. Bioinformatics analysis was conducted, and 256 upregulated genes and 490 downregulated genes were found in GSE125989, 675 upregulated 557 downregulated genes were found in GSE100534 (Fig. 1A), and 79 common genes between two datasets were found in both datasets (Fig. 1B). Heatmap plots of the hub genes in two datasets are shown in Fig. 1C.

With the genes, GO (gene oncology) pathway and KEGG (Kyoto Encyclopedia of Genes and Genomes) pathway analyses were conducted.

This shows that extracellular matrix/structure organization plays an important role in the brain metastasis of breast cancer. Collagen content and binding also participated in the metastasis process (Fig. 1D–. E, Fig. 1F).

**Fig. 2:**
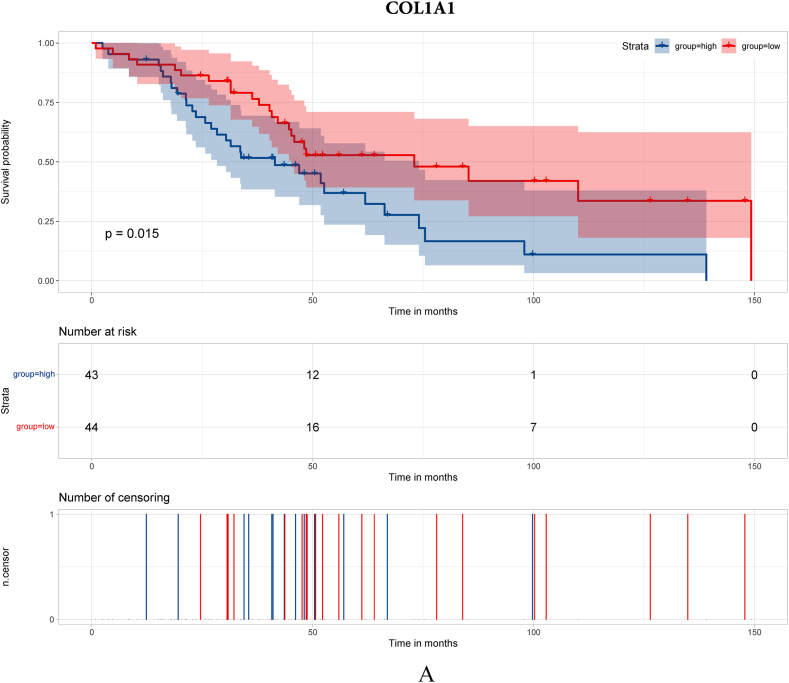

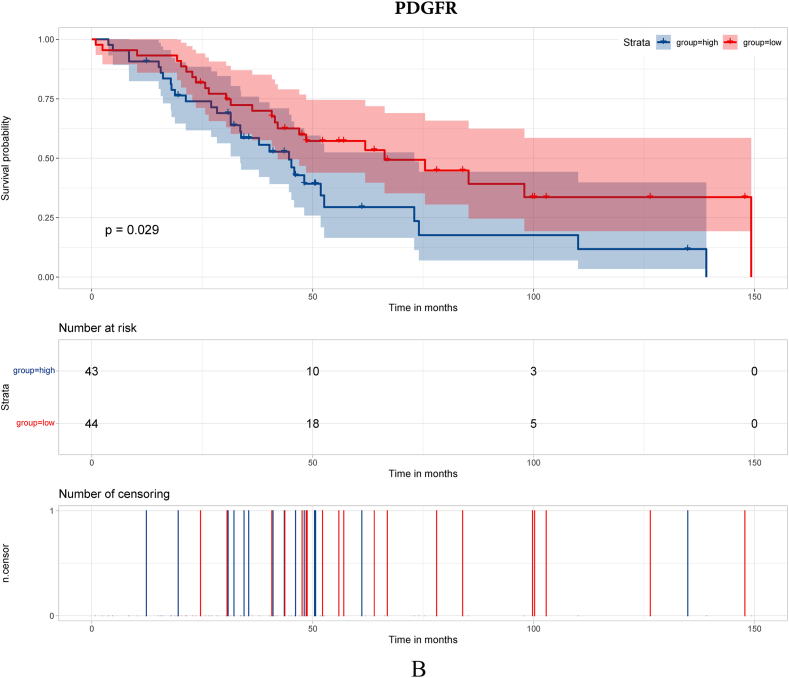

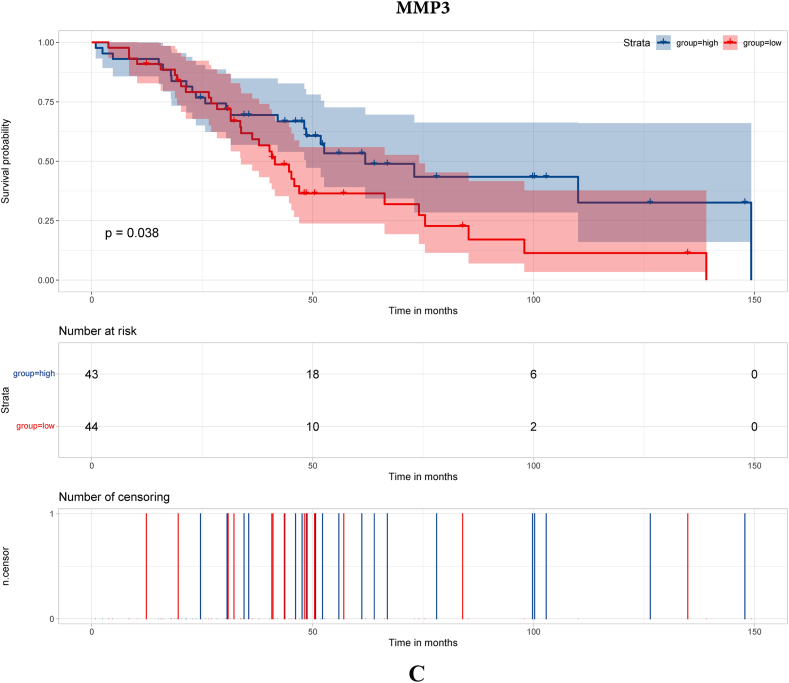

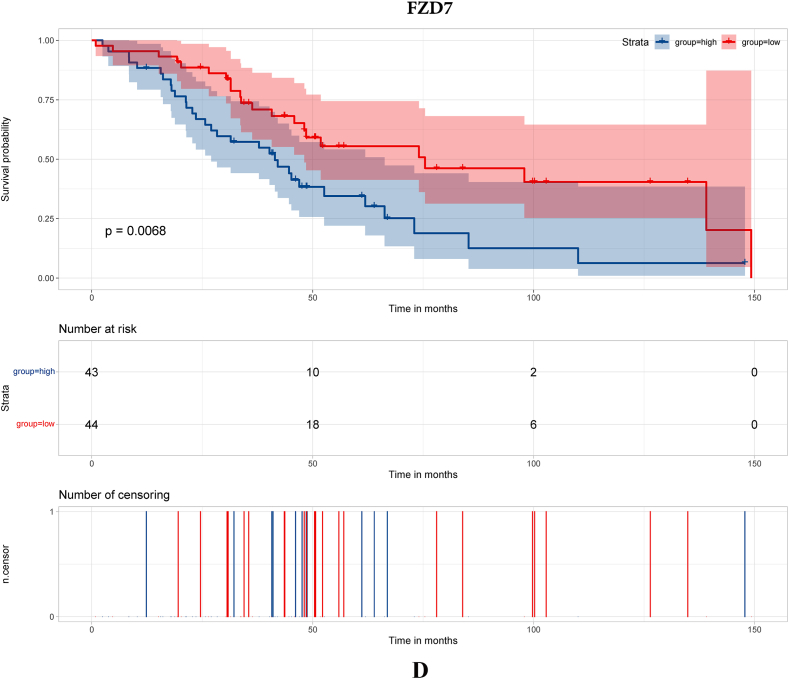
Survival analysis of hub genes. Fig. 2A Survival analysis of COL1A1; Fig. 2B Survival analysis of PDGFR; Fig. 2C Survival analysis of MMP3; Fig. 2D Survival analysis of FZD7.

To search for hub genes, GSE38057 was included for survival analyses. Seventy-nine genes were included in the survival analyses with survival data in GSE38057. The results demonstrated that COL1A1, PDGFR, MMP3 and FZD7 were related to survival (Fig. 2A–D, respectively).

**Fig. 3:**
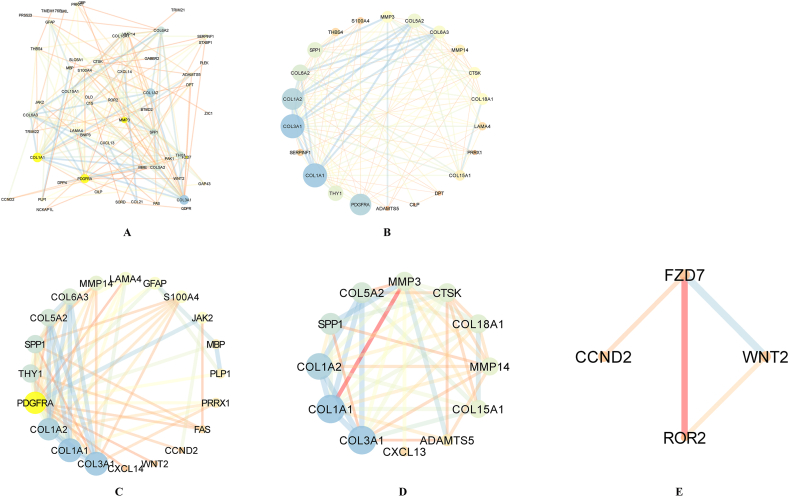
Protein-protein interaction of hub genes. Fig. 3A Protein-protein interaction of 79 hub genes; Fig. 3B Protein-protein interaction of COL1A1 and related-genes; Fig. 3C Protein-protein interaction of PDGFR and related-genes; Fig. 3D Protein-protein interaction of MMP3 and related-genes. Fig. 3E Protein-protein interaction of FZD7 and related-genes.

Protein‒protein interaction analysis was also conducted, and we uploaded the 79 genes into the STRING database to build the PPI network, which was reformed in Cytoscape (Fig. 3A). Moreover, we divided the PPI network into four parts according to the four hub genes, Result demonstrated that the four genes could establish close ties in many ways, which show that the four genes may in some pathways. (Fig. 3B–E).

**Fig. 4:**
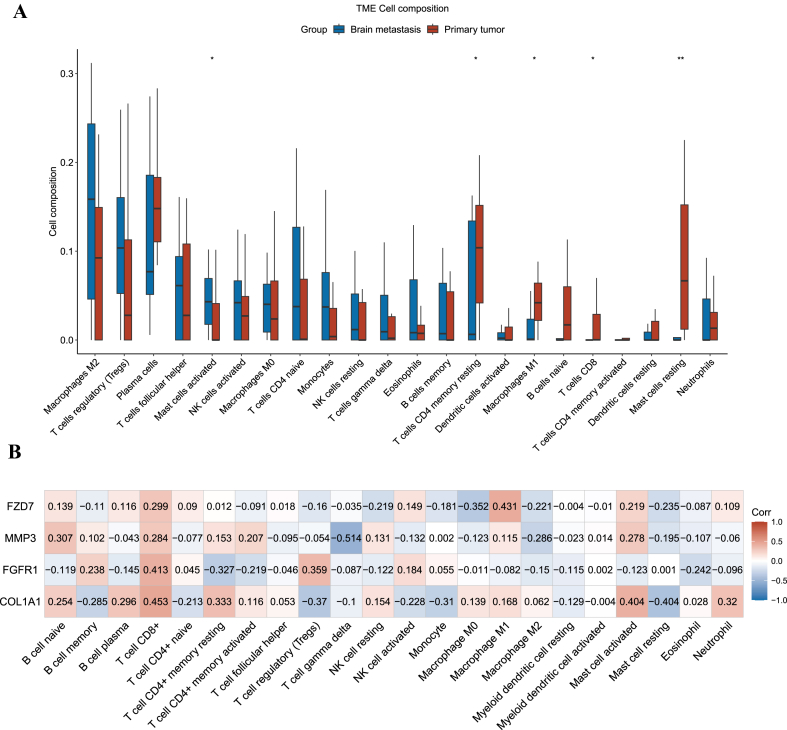
Immune cell infiltration between PBC and BCBM. Fig. 4A Immune cell infiltration between PBC and BCBM; Fig. 4B Relationship of immune cell infiltration and hub genes expression in BCBM.

To disclose the immune microenvironment of brain metastasis of breast cancer, immune cells in the tumor microenvironment were detected in primary and brain metastasis of breast cancer. And GSE125989 was used to analyze the immune infiltration between BC and BCBM. The results showed that CD8^+^ T cells, macrophages (M1), CD4 memory resting T cells and resting mast cells were reduced in the brain metastasis of breast cancer (Fig. 4A). Correlations between hub gene expression and the immune microenvironment were also determined, and the results showed that hub gene expression was positively correlated with CD8^+^ T cells (Fig. 4B), which demonstrated that hub gene expression may be correlated with variations in the tumor microenvironment.

**Fig. 5:**
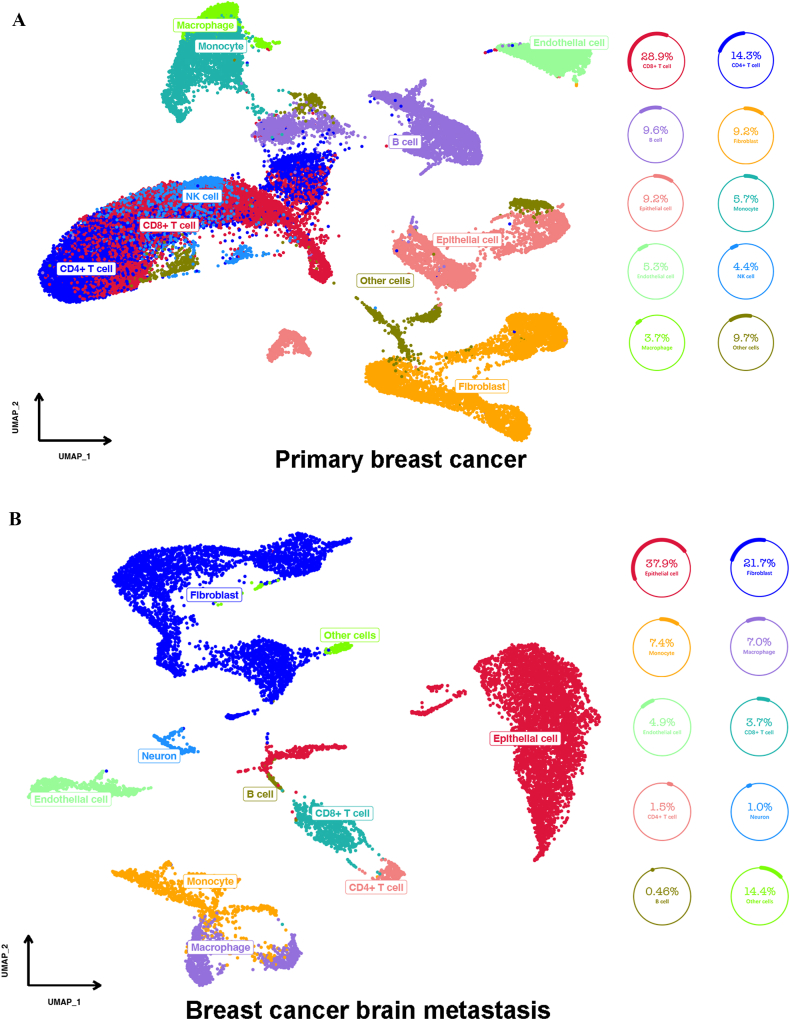
UMAP plot displaying the tumor microenvironment of PBC and BCBM. Fig. 5A UMAP plot displaying the tumor microenvironment of PBC; Fig. 5B UMAP plot displaying the tumor microenvironment of BCBM.

Further analyses of single cells were also conducted, and two datasets (GSE176078 for primary breast cancer and GSE186344 for breast cancer brain metastasis) were included (Fig. 5). The umap plot showed that primary breast cancer was enriched with CD8/CD4+ T cells (28.9 % and 14.3 %), B cells (9.6 %) and CAFs (9.2 %) (Fig. 5A), while breast cancer brain metastasis was enriched with CAFs (21.7 %) and epithelial cells (37.9 %), while CD8^+^ T cells (3.7 %), CD4^+^ T cells (1.5 %) and B cells (0.46 %) were significantly decreased (Fig. 5B). Moreover, in patients with breast cancer brain metastasis, NK cells increased in primary breast cancer (4.4 %) compared with breast cancer brain metastasis (2.0 %)

**Fig. 6:**
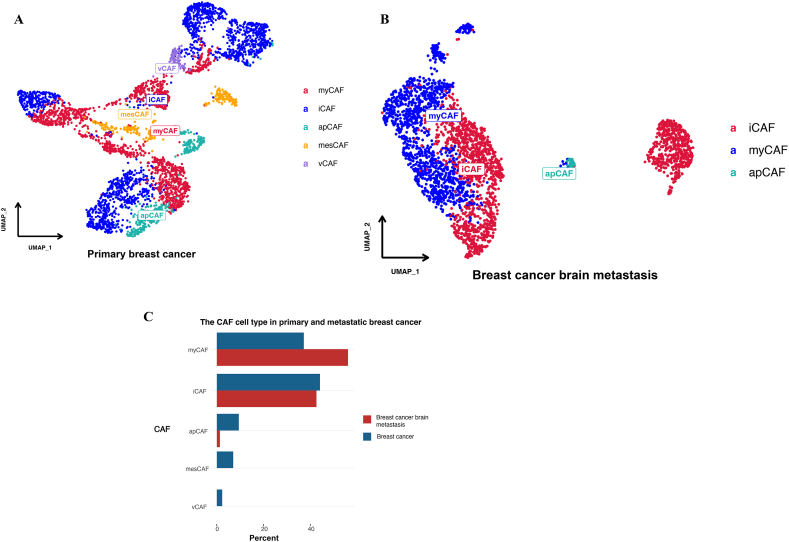
UMAP plot displaying the cancer-associated fibroblasts of PBC and BCBM. Fig. 6A UMAP plot displaying the cancer-associated fibroblasts of PBC; Fig. 6B UMAP plot displaying the cancer-associated fibroblasts of BCBM; Fig. 6C The proportion of cancer-associated fibroblasts between PBC and BCBM.

The GO and KEGG analyses showed that focal adhesion was a major part of the tumor metastasis process in breast cancer, and single-cell analyses showed that CAFs play an important role in the microenvironment of the primary and metastatic sites of breast cancer. Further analyses of CAFs were conducted (Fig. 6). The results demonstrated that in primary breast cancer, apCAFs were higher than in breast cancer brain metastasis (7 % vs 1 %), and myCAFs were lower than in breast cancer brain metastasis (36 % vs 57 %) (Fig. 6A–C).

**Fig. 7:**
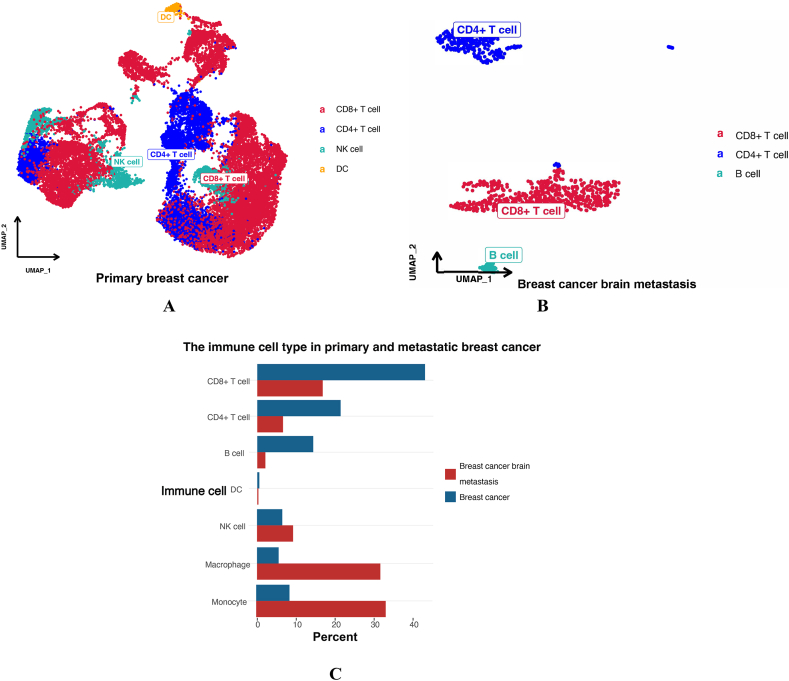

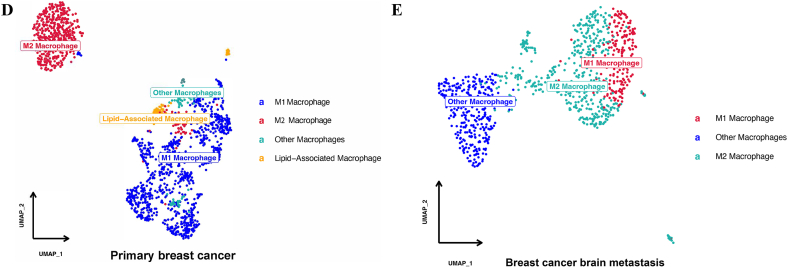
UMAP plot displaying the immune microenvironment of PBC and BCBM. Fig. 7A UMAP plot displaying the immune microenvironment of PBC; Fig. 7B UMAP plot displaying the immune microenvironment of BCBM; Fig. 7C The proportion of immune cells between PBC and BCBM; Fig. 7D UMAP plot displaying the macrophages of PBC; Fig. 7D UMAP plot displaying the macrophages of BCBM.

Immune microenvironment analysis was also conducted in primary and metastatic breast cancer (Fig. 7). The results showed that the proportions and amounts of CD8^+^ T cells and CD4^+^ T cells were higher in primary breast cancer than in breast cancer brain metastasis (Fig. 7A,7B). The number of dendritic cells was higher in primary breast cancer (Fig. 7C). In breast cancer brain metastasis, macrophages and monocytes were significantly higher than those in primary breast cancer(Fig. 7D,7E). The type of macrophages differed between primary breast cancer and breast cancer brain metastasis: M1 macrophages were the major type in primary breast cancer, while M2 macrophages were the major type in breast cancer brain metastasis.

## 4 Discussion

In this study, we enrolled public datasets to illustrate the difference in gene expression in primary and brain metastases of breast cancer. We found that COL1A1, MMP3, PDGFR and FZD7 were significantly increased/decreased in breast cancer brain metastasis compared with primary breast cancer, and the four genes were also acted as long-term outcome factors in breast cancer brain metastasis. Moreover, we further explored the microenvironment of primary breast cancer and breast cancer brain metastasis. The results showed that immune-respond cells play a major role in primary breast cancer, while CAFs and immunosuppression cell play a major role in breast cancer brain metastasis. Then, we probed the immune microenvironment and CAF classification in primary breast cancer and breast cancer brain metastasis. We found that the proportions of CD4^+^ T cells, CD8^+^ T cells and apCAFs were significantly higher in primary breast cancer, which demonstrates that the immune microenvironment may play an important role in the breast cancer metastasis process.

The extracellular matrix (ECM) is a vital component of the tumor microenvironment and plays an important role in tumor invasion and metastasis [14], and COL1A1 was proven to be a pro-metastatic ECM gene, which is believed to be a poor long-term survival factor in breast cancer [15,16]. Although Fang et al. [15]. Reported that COL1A1 was significantly decreased in breast cancer brain metastasis, they did not explore the long-term impact of COL1A1 in breast cancer brain metastasis. In this study, we found that COL1A1 was a poor predictor for long-term survival of breast cancer brain metastasis, and oncology analyses showed that focal adhesion was a major biological process in which COL1A1 participated. The PPI network showed the same result as the biological process. Moreover, we further analyzed the expression of COL1A1 in primary breast cancer and metastatic lymph nodes of breast cancer and showed that COL1A1 was higher in metastatic lymph nodes than in primary breast cancer.

MMP3 is a member of the MMP family, which is believed to play an important role in tumor invasion and metastasis [17]. Wolburg et al. [18] found that MMP3 was associated with a breakdown of the blood‒brain barrier (BBB) and increased the risk of tumor metastasis. Moreover, MMP3 expression was associated with the enrichment of tumor-associated macrophages (TAMs) [19], which are believed to be essential in the development of melanoma brain metastasis. In this study, MMP3 was significantly decreased in breast cancer brain metastasis, and the single-cell analysis showed that the breast cancer brain metastasis sample was enriched with macrophages. The above result indicated that MMP3 contributed to the metastasis process of breast cancer. PDGFR is a hot gene in both fundamental research and target-medicine development. PDGFR is believed to play an important role in tumor metastasis and was set as a targeted site in cancer therapy [20]. Turrell et al. [21] found that PDGF can increase the expression of fibroblast activation and fibrosis genes and was associated with the metastasis process in breast cancer.

The tumor microenvironment (TME) of breast cancer was introduced in previous studies [22,23], while the TME in breast cancer brain metastasis is poorly understood. In this study, we compared the primary and brain metastasis of breast cancer with single-cell data. The results demonstrated that primary breast cancer samples were rich in immune cells, while the brain metastatic samples were rich in fibroblasts. This indicated that the immune microenvironment of breast cancer brain metastasis was worse than that of primary breast cancer. Risom et al. [22]. Found that the metastasis process was coordinated with changes in the TME, especially the proportions of fibroblasts and immune cells. Noh et al. [24] also found that immune cell populations were significantly decreased in breast cancer brain metastasis.

Further analysis of immune cells was conducted. Compared with primary breast cancer, breast cancer brain metastasis was short of CD8^+^ T cells and CD4^+^ T cells, while the macrophages and monocytes were higher than those in primary breast cancer. Liu et al. found that T-cell proliferation was suppressed in metastatic breast cancer compared with primary breast cancer, and CD4^+^ T cells were more likely to differentiate into exhausted cells in metastatic breast cancer. Huang et al. [25] disclosed that M2 macrophages play an important role in tumor metastasis, and CD2/CD27 inhibited the activation of nitrogen metabolism pathways and suppressed M2 polarization of macrophages. All the results mentioned above showed that immunosuppression plays a leading role in the tumor microenvironment of breast cancer brain metastasis. Nayyar et al. [26] also found the different properties of intracranial tumor with extracranial tumor, and the benefit of combination CDK4/6 and PD-1inhibition was proved.

Cancer-associated fibroblasts (CAFs) are a major component of the TME and are classified into two categories: myofibroblast CAFs (myCAFs) and inflammatory CAFs (iCAFs). CAFs are regarded as crucial regulators of the TME [27], and previous studies have proven that myCAFs can increase the stiffness of the extracellular matrix and protect tumors from cytotoxic T cells, effector T cells and dendritic cells, while iCAFs attract myeloid-derived suppressors, M2 macrophages and regulatory T cells [28,29]. Song et al. [30] demonstrated that CAF-derived cardiotrophin-like cytokine Factor 1 increased the expression of CXCL6 and TGF-β, which increased cancer stem cells, advanced the tumor stage and led to a poor prognosis. Grauel et al. [31] found that although myCAFs promoted tumor stages and deteriorated long-term outcomes, after remodeling CAF dynamics by neutralization of TGF-β, CAFs develop productive antitumor immunity and better efficacy of PD-1 immunotherapy, especially antigen-presenting CAFs (apCAFs).

Elyada et al. [9] first introduced apCAFs in pancreatic cancer in 2019, which are characterized by noncanonical expression of the MHC class II complex. They found that apCAFs were associated with immunosuppression and poor long-term outcomes. However, Toulmin et al. [32] found that apCAFs contributed to lung adaptive immune responses without a hyperinflammatory response. Friedman et al. [10] also found that apCAFs correlated not only survival but also breast cancer mutations, which lead to DNA damage and increased somatic mutational load and immune cell infiltration. In this study, we found that apCAFs were correlated with CD8^+^ and CD4^+^ T cells, indicating that in breast cancer, apCAFs play a positive role in the suppression of tumor growth and metastasis.

There are some limitations of this study. Firstly, since the samples of breast cancer brain metastasis were hard to access, further certified data were not provided in this study. A further experiment will be conducted in the future. Secondly, all the datasets were from public database, the publication bias and the selection bias would impact the result in this manuscript, more samples were need to verify the conclusion.

## 5 Conclusion

In this study, we uncovered the differences in gene expression and the tumor microenvironment of PBC and BCBM. First, we analyzed gene expression in PBC and BCBM and found hub genes (COL1A1, PDGFR, MMP3 and FZD7) between primary and brain metastatic breast cancer. GO and KEGG analyses found that focal adhesion plays a vital role in the metastasis process. Further analysis of single-cell data showed that BCBM was short of immune cell infiltration, consisting largely of CAFs. Further analysis of CAFs found that apCAFs were associated with immune cell infiltration in PBC, while in BCBM, only a small proportion of apCAFs and T cells were associated. These results demonstrate that CAFs play a vital role in the brain metastasis process of breast cancer and provide a foundation for molecular targeted therapy for BCBM. More researches should be conducted to elaborate the mechanism and obtain new treatment strategy of BCBM.

## Disclosure statement

The author reports no conflicts of interest in this work. This study was performed according to the Declaration of Helsinki.

## CRediT authorship contribution statement

**Jingrong Li:** Writing – original draft, Visualization, Funding acquisition, Data curation, Conceptualization. **Nanping Lin:** Writing – original draft, Methodology, Investigation, Funding acquisition, Formal analysis, Conceptualization. **Shengcen Zhang:** Software, Resources, Project administration, Investigation, Funding acquisition. **Lihong Weng:** Visualization, Validation, Project administration, Methodology, Investigation. **Chen Chen:** Software, Resources, Methodology, Investigation. **Wensi Ou:** Validation, Supervision, Resources, Investigation. **Yingping Cao:** Writing – review & editing, Validation, Conceptualization.

## Declaration of competing interest

The authors declare that they have no known competing financial interests or personal relationships that could have appeared to influence the work reported in this paper.
